# A retrospective analysis of adverse events reported by Tunisian intensive care units’ professionals

**DOI:** 10.1186/s12913-024-10544-9

**Published:** 2024-01-16

**Authors:** Mohamed Ayoub Tlili, Wiem Aouicha, Nikoloz Gambashidze, Asma Ben Cheikh, Jihene Sahli, Matthias Weigl, Ali Mtiraoui, Souad Chelbi, Houyem Said Laatiri, Manel Mallouli

**Affiliations:** 1 University of Sousse, Faculty of Medicine of Sousse, Department of Family and Community Medicine, LR12ES03, 4002 Sousse, Tunisia; 2 Institute for Patient Safety, University Hospital Bonn, Venusberg-Campus-1, 53127 Bonn, Germany; 3 Department of Prevention and Care Safety, Sahloul University Hospital, 4054 Sousse, Tunisia

**Keywords:** Adverse events, Reporting and learning systems, Intensive care

## Abstract

**Introduction:**

Adverse events (AEs) that occur in hospitals remain a challenge worldwide, and especially in intensive care units (ICUs) where they are more likely to occur. Monitoring of AEs can provide insight into the status and advances of patient safety. This study aimed to examine the AEs reported during the 20 months after the implementation of the AE reporting system.

**Methods:**

We conducted a retrospective analysis of a voluntary ICU AE reporting system. Incidents were reported by the staff from ten ICUs in the Sahloul University Hospital (Tunisia) between February 2020 and September 2021.

**Results:**

A total of 265 reports were received, of which 61.9% were deemed preventable. The most frequently reported event was healthcare-associated infection (30.2%, *n* = 80), followed by pressure ulcers (18.5%, *n* = 49). At the time of reporting, 25 patients (9.4%) had died as a result of an AE and in 51.3% of cases, the event had resulted in an increased length of stay. Provider-related factors contributed to 64.2% of the events, whilst patient-related factors contributed to 53.6% of the events. As for criticality, 34.3% of the events (*n* = 91) were unacceptable (c3) and 36.3% of the events (*n* = 96) were ‘acceptable under control’ (c2).

**Conclusions:**

The reporting system provided rich information on the characteristics of reported AEs that occur in ICUs and their consequences and may be therefore useful for designing effective and evidence-based interventions to reduce the occurrence of AEs.

## Introduction

Improving patient safety has become a priority worldwide as there has been growing concern about the high prevalence of adverse events (AEs) and medical errors in healthcare settings and their preventable nature [1]. A comprehensive meta-analysis has shed light on the prevalence of AEs within hospital settings, revealing an average of 12% [2]. Also, a scoping review, encompassing 25 studies conducted across 27 countries across six continents, reported that 10% of patients experienced at least one AE (range: 2.9–21.9%) with a median of 7.3% (range: 0.6–30%) of these AEs proved fatal. Furthermore, the analysis indicated that a substantial proportion (between 34.3% and 83%) of these AEs were deemed preventable, implying potential for implementing impactful improvement measures [3]. In Intensive Care Units (ICUs), because of the medical conditions of critically ill patients and the complexity of the clinical procedures, AEs are more likely to occur and to result in severe consequences posing a serious threat to patient safety [4]. Roque et al. [5] reported an incidence of AEs that varied between 0.87% and 34.7%. Because of their poor infrastructure and limited resources, developing countries face an even more dire predicament [6]. In Tunisia, the overall incidence of AEs in surgery departments was 18.1%, of which 62% were considered preventable [7]. Another study revealed that the incidence of AEs in a university hospital was of 12.4%, with hospital acquired infection and unplanned readmission were the most common AEs [8]. In ICUs, Letaief et al’s study [9] showed that 41.1% of patients admitted to ICUs suffered from at least one AE and 70% of these were preventable.

It has been increasingly recognized that reporting systems are key to enhance quality of care and patient safety outcomes [10, 11]. Reporting systems are important to learn from previously committed mistakes, allowing an understanding of the extent and the nature of errors [10–12]. After reporting errors, their root causes can be analyzed, and proper preventive strategies can be implemented to avoid their recurrence [10–12]. International studies have demonstrated how incident reports lead to a better understanding and prevention of AEs in hospital settings [12, 13]. Thus, it is admitted that incident reporting promotes patient safety [14]. To be effective improvement tools, reporting systems have to be supported by a safety culture that promotes learning from errors rather than blaming and shaming individual performances [15].

In Tunisia, reporting systems remain uncommon in hospitals and reporting culture is very little developed. This is demonstrated through the patient safety culture surveys carried out in ICUs and where the dimensions related to frequency of events reported and non-punitive response to error had the lowest scores [16, 17]. There is no national reporting system, nor a specific regulation relating to the implementation of reporting systems in hospitals. The only experience with reporting is the Maternal Mortality system that was initiated in year 2000 in Tunisia. As in many developing countries, this causes lack of data on the nature of AEs, thereby missing learning opportunities and limiting opportunities for quality improvement. Aware of the importance of reporting systems, particularly in ICUs, the Sahloul University Hospital (Sousse, Tunisia) decided to implement an ICU-reporting system in the year 2020.

This study aimed at examining the AEs reported during the first 20 months of the reporting system implementation.

## Methods

### Study design 

We conducted a retrospective analysis of a voluntary ICU-AE reporting system for events reported between February 2020 and September 2021 as the reporting system was implemented in February 2020. The implementation started with an awareness phase, including the training sessions for ICU staff. After the trainings, ongoing awareness-raising activities were carried out, including follow-up trainings and distribution of brochures and posters. Feedback was an integral component of the system, with a response to report form and an annual report.

### Study setting

We analyzed events that were reported by staff from ten ICUs in the Sahloul University Hospitals (Tunisia). The capacity of Sahloul Hospital was 690 beds, with 297,082 consultations and 26,119 admissions recorded in 2016.

For the participating ICUs, the number of beds ranged between 4 and 19, the mean occupancy rates ranged from 47 to 100%, mean length of stay ranged between 6.33 days (± 9.45) to 26 days (± 14.11), and the Nurse per bed ratio ranged between 0.66 and 1.5.

### Data collection

This study reviewed the AEs submitted to the Adverse Events Managements Committee which consisted of fixed committee members which were doctors, nurses and healthcare technicians belonging to the Prevention and Safety Care Department of Sahloul University Hospital. All committee members had a background and expertise in quality of care and healthcare risk management. During the analysis meetings, ICU specialists and any other professionals concerned by the reported events were also invited. The reporting form, that was inspired from the Intensive Care Unit– Safety Reporting System (ICU-SRS) [18], gathered information on the event’s circumstances (date, unit), a description of the event, patient-related information (if the event concerned a patient) such as gender, current treatment and medical record’s number, the consequences of the event (death, length of stay), and the possible leading causes of the event. Disclosure of identity was optional to avoid creating a fear of identification and punishment among reporters.

At first, the reporting was in paper format, then, to adapt to the COVID-19 circumstances, we created an interactive digital form that allowed staff to fill in and submit the reports with their computer/tablet/phone and receive feedback via email.

### Definitions

An adverse event is defined as “a situation which deviates from the procedures or results expected in a usual situation and which is or could potentially be a source of damage” [19]. It can be an accident (“event that has unintentionally happened, that results in damage, injury or harm”), an incident (“unexpected event that does not result in serious losses or injury”), or a near-miss (“unexpected event, that does not result in an injury/illness or damage but had potential to do so”) [19, 20].

Contributing factors are those that contributed to the occurrence of the AE and can be of various types. These include patient related factors which refer to the clinical or social characteristics of a patient that contribute to an AE (e.g. personal beliefs, values, religious taboos, etc.), provider-related factors (e.g. lack of skill in performing the procedure, fatigue), tasks-related factors (e.g. absence of protocol to guide therapy, difficult task to accomplish), team-related factors (e.g. perceived barrier for speaking up, inadequate team structure and leadership), training- and education-related factors (e.g. new staff member or intern performing unfamiliar/difficult task and not seeking help), equipment-related factors (e.g. lack of routine maintenance, poor storage conditions), environmental factors (e.g. full bed census with staff shortage), and institutional factors which refer to the elements, decisions or characteristics of hospital management or departmental management that contribute to an AE (e.g. limited financial resources) [18].

The identification of contributing factors was carried out according to the ALARM method (Association of Litigation and Risk Management), with minor changes to align with the ICU-SRS original sheet. According to this risk analysis method, AEs are analyzed following four steps: At first, the chronology of the facts that led to the event is reconstructed. The second step consists of identifying care defects and deviations from the norm. Afterwards, in the third step, and for each care defect identified, an analysis and identification of the root causes that contributed to its occurrence. Finally, corrective measures are proposed [21, 22].

The criticality of an event (C) corresponds to the product of “F: frequency (from 1: very rare to 5: very frequent) × S: seriousness (from 1: minor to 5: catastrophic)” of the AE [21]. It allows the prioritization of AEs and to classify them by Acceptable events C1 (C = 1 to 6, priority 3), Acceptable under control C2 (C = 8 to 12, priority 2), and Unacceptable events C3 (C = 15 to 25, priority 1) [21].

Based on Adverse Events Management Committee members’ judgment, preventability was assessed (5 - No evidence of preventability; 4 - Minimal possibility of preventability; 3 - Moderate possibility of preventability; 2 - High possibility of preventability; 1 - Total evidence of preventability). AEs with a score of ≤ 3 points were considered preventable. The judgment is based on the assumption, that standard care would have reasonably prevented the AE.

Concerning patient’s death, the reporters, at first, mention that at the time of the report, the patient is deceased. Afterwards, an investigation and analysis involving the different professionals were launched to provide confirmation.

### Data analysis

Data were analyzed using SPSS 26 software (IBM, SPSS Inc., Chicago, IL, USA). Descriptive statistics including frequency (n) and percentage (%) were used for qualitative variables. Differences between different subgroups were explored using Chi-square test. The level of significance was set at 5%.

### Ethical considerations

This research was approved by the Institutional ethics committee of the Faculty of Medicine of Sousse. The ICUs head-chiefs were informed about the study and gave their permission to include reports from their units. The identity of the reporters and patients were kept anonymous.

## Results

### Number and types of AEs

**Fig. 1:**
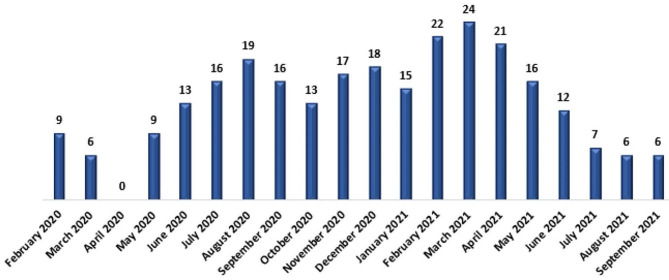
: Number of AEs per month (*n* = 265)

A total of 265 reports were received, of which, 239 (90.2%) were filled in by nursing staff and 102 reports (38.5%) were received via emails (Table 1). During the first month of the implementation, nine events were reported. In March 2021, 24 reports were received (Fig. 1). The most frequently reported AE was healthcare-associated infection (30.2%, *n* = 80), followed by pressure ulcers (18.5%, *n* = 49). Also, 44 AEs was classified under ‘Equipment and material problems’. These AEs concerned monitor inaccuracies (*n* = 12), ventilator malfunctions (*n* = 9), infusion pump errors (*n* = 8), the absence of equipment (*n* = 8), and equipment breakdowns with the absence of spare parts (*n* = 7). Table 2 presents the types of reported AEs.

**Table 1:** Data related to the reporting system outcomes (*n* = 265)

Reporting system related data	n	%	p value
**Reporter grade**			
Physician	26	9.8	< 0.0001
Nursing staff	239	90.2	
**Reception mean**			
Paper-based	163	61.5	0.0003
Via emails	102	38.5	
**At the time of reporting, the patient had died as a result of the event**			
Yes	25	9.4	< 0.0001
No	240	90.6	
**The event prolonged the patient’s hospital stay**			
Yes	136	51.3	0.672
No	129	48.7	
**The event involved a medication error**			
Yes	14	5.3	< 0.0001
No	251	94.7	
**The patient/legal guardian has been informed of the event**			
Yes	49	18.5	< 0.0001
No	216	81.5	
**Preventability**			
Preventable	164	61.9	0.0002
Non-preventable	101	38.1	

**Table 2:** Types of events reported (*n* = 265)

Types of events	n	%
Healthcare associated infections	80	30.2
Pressure ulcers	49	18.5
Equipment and material problems (lack, breakdown, malfunction)	44	16.6
Extubation	19	7.2
Unplanned absence of staff	12	4.5
Patient fall	10	3.8
Blood exposure accident	8	3.0
Medication error	7	2.6
Staff aggression	7	2.6
Organization (e.g. absence of sterile patient circuit)	4	1.6
Transfusion accident	4	1.6
Vein thrombosis	4	1.6
Hygiene/environment (e.g. presence of insects)	3	1.1
Allergic reaction	3	1.1
Venite	2	0.7
Extravasation	2	0.7
Restoration (e.g. diet unrespected)	2	0.7
Others	5	1.9
*Hematoma*		
*Forgetting tourniquet for more than 45 min*		
*Septic dressing remained unchanged for more than a week*		
*Alteration of the respiratory state of a patient following an error in adjusting the breathing parameters*		
*Volkmann’s syndrome following a too tight circular plaster cast*		
**Total**	**265**	**100**

### Preventability and criticality of the AEs

**Fig. 2:**
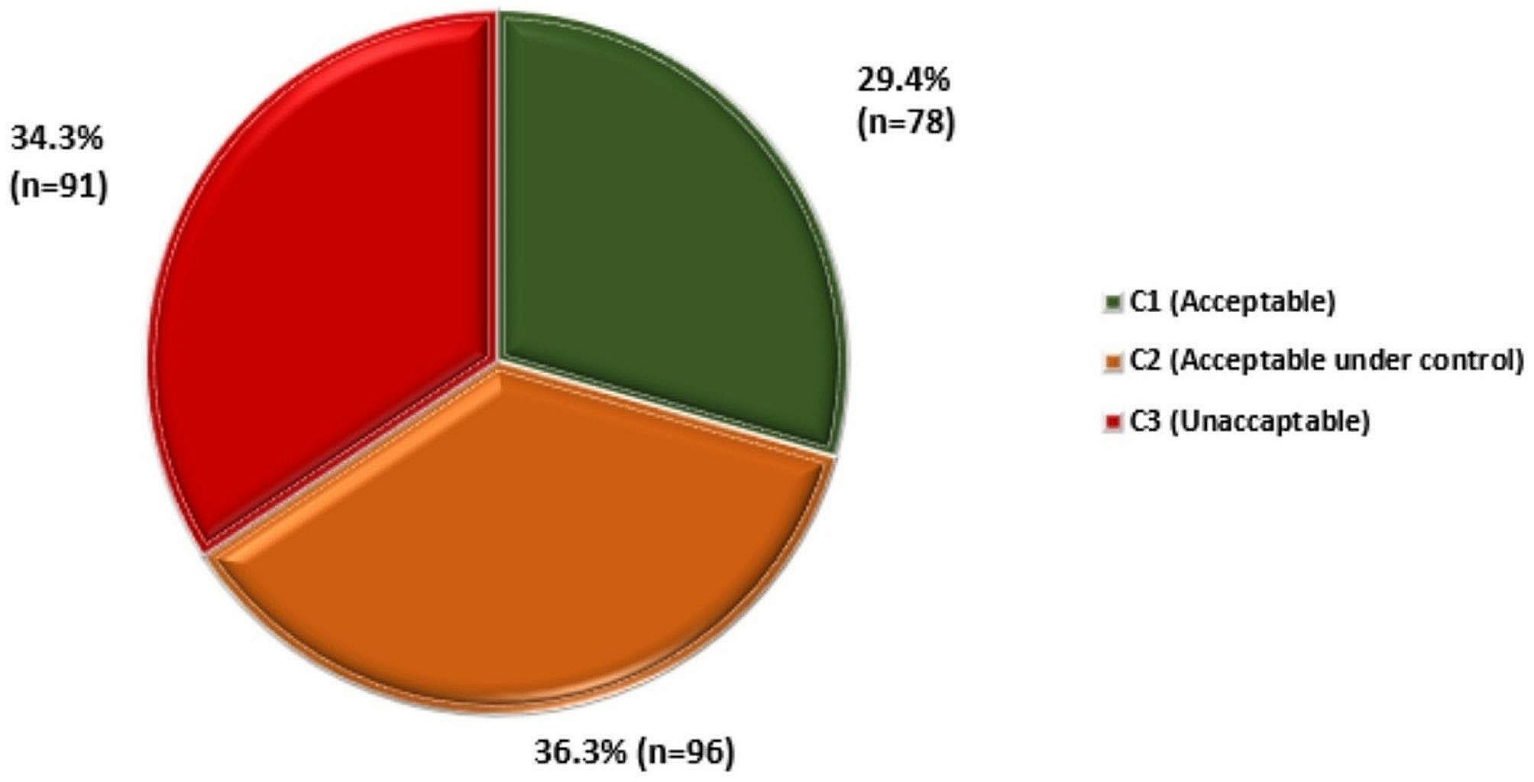
: Criticality of the AEs (*n* = 265)

The majority of AEs (*n* = 164, 61.9%) were deemed preventable. For 46 events (17.4%), there was total evidence of preventability. A judgement of a high possibility of preventability was made for 62 events (23.4%) and of a moderate possibility of preventability for 56 events (21.1%). In contrary, 101 events were deemed non preventable (38.1%), with 61 events (23.0%) having a minimal possibility of preventability and 40 events (15.1%) with no evidence of preventability. As for criticality, 34.3% of the events (*n* = 91) were unacceptable (c3) and 36.3% of the events (*n* = 96) were acceptable under control (c2). For frequency, 38 of the reported AEs (14.3%) were very rare, 33 (12.5%) were rare, 54 (20.4%) were little frequent, 71 (26.8%), occurred frequently and 69 (26.0%) very frequently. As for seriousness of the AEs, 55 (20.8%) were minor, 75 (28.3%) were moderate, 78 (29.4%) were serious, 44 (16.6%) were critical and 13 (4.9%) were catastrophic. Distribution of criticality of AEs is presented in Fig. 2.

### Consequences and contributing factors

At the time of reporting, 25 patients (9.4%) had died as a result of the event and in 51.3% of cases, the event had resulted in an increased length of stay. Provider-related factors contributed to 64.2% of the events, whilst patient-related factors contributed to 53.6% of the AEs (Table 3).

**Table 3:** Contributing factors and consequences of the reported AEs (*n* = 265)

**Contributing factors**	n	%
Provider-related factors	170	64.2%
Patient-related factors	142	53.6%
Team-related factors	136	51.3%
Institutional-related factors	136	51.3%
Environmental-related factors	131	49.4%
Training and education-related factors	118	44.5%
Equipment-related factors	98	37.0%
Tasks-related factors	89	33.6%
**Consequences**	n	%
Death	25	9.4
Increased length of stay	136	51.3
Unplanned surgery	49	18.5
Patient/ accompanying dissatisfaction	161	60.8
Change of care protocol/ medical treatment	90	34
Impairment	27	10.2
Permanent impairment	12	4.5
Additional test	97	36.6

### Treatments received

The ongoing treatments that patients were undergoing at the time of documenting adverse events (not in response to the AE) were: central venous catheter (90.2%), bladder catheterization (89.8%), sedation (87.9%), mechanical ventilation (86.8%), inotropes (86.8%), invasive blood pressure monitoring (70.2%), multiple infusions (60.8%), thoracic drainage (45.3%), monitoring intracranial pressure (40.0%), restraint (29.8%), **dialysis (14.0%)**, suprapubic catheter (10.2%), and pulmonary artery catheter (9.1%).

## Discussion

The high frequency of AEs in healthcare settings, as well as their avoidable nature, have made improving patient safety and quality of care, particularly in ICUs, a global priority. Given the magnitude of the problem, AEs reporting systems are acknowledged as being crucial in identifying and gaining insights from these AEs.

Remarkably, comprehensive analyses of AEs within ICUs, particularly in middle and lower-income countries, remain scarce within the existing literature. The scarcity of such investigations in these contexts is a cause for concern, as patient safety is often more threatened due to resource limitations, unique healthcare system challenges, and varying levels of access to advanced technologies and training. Our study fills this critical gap by conducting a retrospective analysis of AEs reported by ICUs professionals working in Sahloul university hospital (Sousse, Tunisia).

In total, we received 259 reporting files. In reality, it is challenging to compare the results of our AEs reporting system with those of other systems throughout the world. Number of reports revealed by articles analyzing reporting system’s outcomes varies according to the duration, the settings included, and the nature of the events taken into account. For articles concerning reporting systems in ICUs, they also vary in terms of the number of ICUs as well as the study duration.

For instance, the study by Thomas et al. [23] in which patient safety incident reports of 30 critical care units were retrospectively analyzed over a period of five years (from 2009 to 2013) revealed that a total of 19,945 reports were received, giving an average of 3989 reports per year. Another study [24], that aimed at analyzing incident reporting system reports in 32 ICUs of ten hospitals in South Africa, reported that in a period of three months, 1017 reports were received. The study by Ilan et al. [11], conducted in 2 adult ICUs reported a total of 332 reports received over a period of one year.

The number of reports received by the end of our intervention may therefore seem not sufficient. However, it is important to admit that our system is still new, “not mature” enough, and insufficiently developed. In fact, all reporting systems struggle with the issue of underreporting, especially throughout the early stages of their adoption, and even after the system is well-established and deployed, only a small proportion of actual events are reported [25]. According to literature, only 7–15% of AEs are being reported [26].

Krouss et al. [27] claimed that despite incident reporting systems being standard in hospitals, reporting is rare. And although the majority of healthcare professionals are aware of the existence of these systems, only very few have actually submitted a report [27]. According to Gqaleni and Bhengu’s analysis of a patient safety incident reporting system in critical care, 16% of the participants had never used it [24]. The causes of underuse included lack of response to reports (4%), fear (9%), and busy schedules (3%) [24].

COVID-19 pandemic may also have played its part in limiting reporting as it coincided with our intervention. In fact, many studies reported the decrease of AEs reporting after the onset of the COVID-19 pandemic [28–32]. Moroto et al. reported an overall decrease in incident reporting of 60.7% [28]. A study that explored incident reporting during the COVID-19 pandemic in a tertiary Italian hospital showed that during COVID-19 pandemic, and especially during the first and second waves, there was a statistically significant reduction in the rate of incident reporting (215 reports per 1000 admissions in 2019 vs. 167.9 reports per 1000 admissions in 2020; *p* = 0.001) [32].

It is recommended that, especially during a healthcare crisis, such as a pandemic, patient safety and quality of care should always remain a priority, and that efforts must enable continuous organizational improvements [32, 33]. AE reporting and learning systems are here extremely important, to figure out what is impacting the safety and whether additional organizational adjustments are needed [30, 33]. During a pandemic event, learning opportunities could be improved by capturing crisis-related incidents [32–34]. Hence, education and training interventions are crucial to raise awareness of the importance of reporting among health workers [32].

The most reported event in our study was healthcare-associated infection (HAI). HAIs pose a significant threat to patient safety particularly in intensive care settings [35]. ICU patients are at higher risk for HAIs, especially in low and middle-income countries, where 30% of the ICU patients are affected by at least one HAI [35]. Furthermore, evidence indicates that, regardless of the nation’s economic status, 35–55% of HAIs could be avoided [36].

The lack of compliance with hand hygiene and other basic Infection Prevention and Control (IPC) measures among healthcare workers in ICUs leads to the cross-infection of microorganisms from a patient to another [37]. The coronavirus pandemic also brought to light the difficulty that an emerging virus presents in modifying prevention strategies regarding both the risk of exposure to healthcare providers and the requirement to preserve the highest level of care [37]. Training and awareness-raising sessions on basic IPC measures is therefore one of the effective initiatives that can be undertaken to prevent and lead to a significant reduction in the incidence of infections in the ICU with resulting reduced health care costs [37].

In response to several reports related to HAIs, the adverse events management committee implemented a set of measures, including training sessions on standard precautions and basic IPCs, hand hygiene, and placement of invasive devices. This was in addition to the annual instantaneous HAI prevalence survey in order to follow the general trend of HAIs. Indeed, surveillance of HAIs, particularly in intensive care settings, allows to improve infection control and healthcare quality [38].

After HAIs, with 49 reports, pressure ulcers (PUs) were the second most reported AE. This was also shown in other contexts, where PU were noted among the top 5 AEs being reported [39]. Patients admitted to ICUs are more likely to develop PUs due to the heightened risks associated with critically ill patients, including circulatory impairment brought on by immobility, hemodynamic instability, vasopressor medication, decreased sensory awareness, and organ failure [39]. A study reported the incidence of PUs in ICUs between 10% and 41% [40].

Education has been identified as a factor that may contribute to the prevention of PUs [39]. In one study, healthcare professionals who took part in a brief educational session (of less than 1 h) showed that it was beneficial in increasing staff awareness of the PU problem and their knowledge of prevention methods [41]. In addition, providing staff with audit feedback has also been cited as a prevention initiative [39, 41]. When clinicians are aware of the findings of analysis, they become more involved in PU prevention [39, 41]. Real-time informal and formal feedback, are useful in raising clinician awareness of PU prevention [41].

As part of the implementation process, in an attempt to respond to the large number of reports related to PUs, we carried out an information and awareness session on the magnitude of the problem of bedsores and on prevention means. Awareness-raising brochures were distributed, which highlighted the magnitude of the PU problem, its prevention and the course of action in case of occurrence. The response form to the report, which was given as feedback, sounded the alarm on this problem and PUs issue was also discussed during patient safety rounds with the unit managers.

Recent research has brought significant insights into the realm of pressure ulcer prevention and treatment. One key finding underscored the effectiveness and cost-efficiency of risk stratification, advocating for the allocation of pressure-injury prevention measures exclusively to patients with low Braden scores [42]. This tailored approach not only minimizes costs but also proves to be more efficacious than conventional care methods. Additionally, exploration of the Quality Function Deployment (QFD) process demonstrated a systematic method for evaluating solutions to major clinical problems, including pressure ulcers. Through QFD, five high-priority targets for future investigation were identified, encompassing automated activity reminders, pressure ulcer risk assessment tools, clinician training, educational videos, and patient pressure maps [43]. These findings highlight the importance of meticulous planning and commitment in the implementation of strategies for enhanced pressure ulcer prevention. Importantly, both of these recommendations are applicable in the Tunisian context, as they are not overly complex and can be easily implemented, offering a valuable approach to improving patient care and reducing healthcare costs.

In our retrospective analysis of AEs, a subset comprising 44 events were related to equipment and material problems. The frequency and variety of equipment-related AEs underscore the multifaceted nature of challenges encountered, emphasizing the significant impact of equipment functioning on patient care within Tunisian ICUs. The prevalence of monitor inaccuracies, ventilator malfunctions, and infusion pump errors highlights the critical need for targeted interventions aimed at enhancing equipment reliability, maintenance protocols, and inventory management practices to mitigate these recurrent issues and bolster patient safety.

Another important finding is that majority of the reports (90.2%) were made by nurses. In fact, this phenomenon is widespread and many other studies reported that incident reporting is especially low among physicians, as compared to nurses [44–46]. Mahajan, in his article, tried to provide an explanation, stating that poor reporting practices by physicians may reflect a deeply rooted belief in medicine, that only bad physicians make mistakes and therefore physicians are ashamed to report AEs [47].

In 51% of cases, the reported AEs were associated with increased length of stay and 9.4% of them were associated with mortality. Indeed, AE occurrence in intensive care has been reported to increase mortality and length of stay in several studies [5]. The impact on the increased lengths of stay and death for patients who experienced AEs in critical care is a severe issue demonstrating the necessity for initiatives focused on improving the quality of care, especially in low- and middle-income countries, where these AEs and lengthened stays are associated with increased treatment costs.

Team-related factors were present in 51.3% of the contributed factors to the reported AEs. Patient care in the ICU particularly requires vigilant synchronization of efforts of different highly qualified clinicians with different knowledge, competencies, and attitudes [48]. As a result, teamwork and communication are crucial for ensuring effective and safe healthcare services in these settings [48]. An association between the level of teamwork and ICU outcomes such as shortened length of stay, lower incidents of periventricular/intraventricular hemorrhage or periventricular leukomalacia, and reduced likelihood of mortality and/or readmission has been reported [48]. Particular emphasis should be placed on teamwork in ICUs to help fight against AEs and promote patient safety outcomes.

The assessment of preventability and criticality within the spectrum of AEs reveals significant implications for patient safety and risk management. Notably, a majority of AEs, accounting for 61.9% of the reported incidents (n = 164), were identified as preventable. This observation emphasizes the need for proactive measures and targeted interventions to avert such incidents, highlighting opportunities for enhancing patient safety within ICUs. Moreover, the criticality categorization unveiled that 34.3% of the events (n = 91) were deemed unacceptable (c3), indicating high-risk incidents requiring immediate attention and systematic improvements in risk mitigation strategies, protocols, and resource allocation to address these critical events effectively. Also, 36.3% of the events (n = 96) fell within the ‘acceptable under control’ category (c2), signifying their serious nature and the imperative need for stringent control and management measures to handle these situations effectively. These findings underscore the urgency for continuous evaluation, refinement of risk management strategies, and comprehensive preventive measures to minimize preventable AEs and effectively manage critical incidents, thereby fortifying patient safety and optimizing care delivery within ICUs.

This study leveraged a comprehensive dataset derived from reported AEs within Tunisian ICUs, providing a rich and a multifaceted examination of AEs, including detailed categorization by type, preventability, and criticality. By focusing on AEs and their contributing factors, the study directly addresses crucial aspects of patient safety within the critical care setting. Furthermore, the study contributes significantly to the limited literature on AEs, especially in middle and lower-income countries and ICUs, providing valuable contributions to the global understanding of patient safety issues in ICU settings.

The insights garnered from this study provide significant implications for clinical practice; by highlighting prevalent AEs, our findings provide a basis for informed decision-making in ICU settings. These insights can prompt the development of tailored protocols, training modules, and quality improvement initiatives aimed at enhancing patient safety and optimizing care delivery. Furthermore, our study underscores the importance of establishing standardized reporting mechanisms for AEs, facilitating proactive strategies to address these issues in real-time, thus fostering a culture of continuous improvement within ICU environments. The implications of our study provide also foundation for future research; it underscores the critical need for further investigations into AEs, offering a template for similar analyses in regions facing comparable healthcare challenges and can therefore stimulate comparative studies across diverse healthcare settings. Also, the identification and categorization of prevalent adverse events, present opportunities for targeted research into interventions aimed at mitigating these issues.

However, this study has some limitations; as the study relies on the retrospective analysis of spontaneous reports of AEs, where some reports incompleteness was observed and some details, such as patient’s medical record reference, were sometimes lacking, limiting further investigation. Additionally, the findings and conclusions derived from this study might have limited generalizability due to inclusion of a unique hospital. Lastly, the data collection process heavily depends on self-reports from healthcare professionals. This introduces the potential for a reporting bias, as individuals may be influenced by their perceptions, experiences, or concerns when reporting AEs.

## Conclusions

The reporting system provided rich information on the characteristics and consequences of reported AEs that occurred in ICUs, and may be therefore useful for designing effective evidence-based interventions to reduce the occurrence of AEs. However, the number of reports over the first 20 months was relatively low and required further supportive efforts. The implementation was possible, even though it coincided with a major constraint like COVID-19 pandemic. Preventive measures should be directed to strengthening the existing strategies and advocating for additional safety measures against AEs.

## Data Availability

On demand, from corresponding author.

## Abbreviations

AE(s): Adverse Event(s)
ICUs: Intensive Care Units
IPC: Infection Prevention and Control
HAIs: Healthcare-Associated Infections
PUs: Pressure Ulcers
QFD: Quality Function Deployment
